# Factors associated with ductal carcinoma in situ (DCIS) treatment patterns and patient-reported outcomes across a large integrated health network

**DOI:** 10.1007/s10549-022-06831-w

**Published:** 2022-12-17

**Authors:** Hayeon Kim, Hong Wang, Kathryn Demanelis, David A. Clump, John A. Vargo, Andrew Keller, Mia Diego, Vikram Gorantla, Kenneth J. Smith, Margaret Q. Rosenzweig

**Affiliations:** 1 Department of Radiation Oncology, UPMC Hillman Cancer Center, Pittsburgh, PA USA; 2 Department of Biostatistics, UPMC Hillman Cancer Center, Pittsburgh, PA USA; 3 Department of Breast Surgery, UPMC Hillman Cancer Center, Pittsburgh, PA USA; 4 Department of Medical Oncology, UPMC Hillman Cancer Center, Pittsburgh, PA USA; 5 Clinical and Translational Science and Center for Research On Health Care, University of Pittsburgh School of Medicine, Pittsburgh, PA USA; 6 Department of Nursing, University of Pittsburgh School of Medicine, Pittsburgh, PA USA; 7 Department of Radiation Oncology, UPMC Hillman Cancer Center, Magee Women’s Hospital, 300 Halket Street, Pittsburgh, PA 15213 USA

**Keywords:** DCIS, Quality of life, Treatment decision-making, Disparity, Breast cancer

## Abstract

**Purpose:**

To examine associations between ductal carcinoma in situ (DCIS) patients’ characteristics, treating locations and DCIS treatments received and to pilot assessing quality-of-life (QoL) values among DCIS patients with diverse backgrounds.

**Methods:**

We performed a retrospective tumor registry review of all patients diagnosed and treated with DCIS from 2018 to 2019 in the UPMC-integrated network throughout central and western Pennsylvania. Demographics, clinical information, and administered treatments were compiled from tumor registry records. We categorized contextual factors such as different hospital setting (academic vs. community), socioeconomic status based on the neighborhood deprivation index (NDI) as well as age and race. QoL survey was administered to DCIS patients with diverse backgrounds via QoL questionnaire breast cancer module 23 and qualitative assessment questions.

**Results:**

A total of 912 patients were reviewed. There were no treatment differences noted for age, race, or NDI. Mastectomy rate was higher in academic sites than community sites (29 vs. 20.4%; *p* = 0.0045), while hormone therapy (HT) utilization rate was higher in community sites (74 vs. 62%; *p* = 0.0012). QoL survey response rate was 32%. Only HT side effects negatively affected in QoL scores and there was no significant difference in QoL domains and decision-making process between races, age, NDI, treatment groups, and treatment locations.

**Conclusion:**

Our integrated health network did not show chronically noted disparities arising from social determinates of health for DCIS treatments by implementing clinical pathways and system-wide peer review. Also, we demonstrated feasibility in collecting QoL for DCIS women with diverse backgrounds and different socioeconomic statuses.

## Introduction

Ductal carcinoma in situ (DCIS) diagnosis rates increased by 500% from 1983 to 2003, largely due to increased mammographic screening, equating to approximately 60,000 DCIS cases annually, about 25% of all new US breast cancers [1, 2]. Overall DCIS incidence has been stable for the last decade, resulting in approximately 1 million women with a history of DCIS. Variation in surgical, radiation, and hormonal therapies exist for DCIS; however, regardless of treatment approach, 10-year cancer-specific survival is above 98% [3–5].

With many treatment options available but no differences in survival, variations in treatment techniques, and decisions may be affected by sociodemographic characteristics or patient and health care provider perceptions [6–9]. DCIS treatment choice in general trends toward overtreatment because less is known regarding de-escalation strategies including opportunities to maintain excellent outcomes while minimizing toxicities and detriments to quality of life [10].

Numerous studies have reported significant disparities in cancer care arising from sociodemographic characteristics and other social determinants of health [11–16]. DCIS has been also reported disparities in outcomes [13, 14]. However, DCIS treatment patterns according to patient characteristics and treatment locations have not been clearly elucidated or assessed.

Health-related quality of life (QoL) is one of essential metrics to assess patient reported outcomes and the benefits of treatments [17, 18]. Studies of QoL, while numerous in breast cancer are scarce in exclusively DCIS populations [19]. There are differences in treatment, prognosis, and survivorship concerns between DCIS and invasive breast cancer, implying that QoL in DCIS women may be unique. Therefore, processes for understanding QoL in patients with DCIS regarding their treatment choices, decision-making, and subsequent QoL assessment are important in terms of value-based care [7].

Herein the present study has two aims: The first one was to examine associations between DCIS patients’ characteristics and DCIS treatments received. The second aim was to pilot collecting QoL among DCIS patients with diverse clinical and sociodemographic backgrounds on their treatment outcomes, decision-making process, and satisfaction of care. The objective of this pilot QoL survey was to ensure that questionnaire data can be obtained from DCIS patients with diverse backgrounds to assess whether these data can be prospectively collected in future work.

## Methods and materials

### Data collection from tumor registry

With University of Pittsburgh Institutional Review Board approval in October 2021, we performed a retrospective tumor registry review of all patients diagnosed and treated with DCIS from 2018 to 2019 in the UPMC network. The data included patients from nine hospitals located within Pittsburgh, including two academic teaching hospitals (academic practice/urban) and ten community hospitals outside Pittsburgh (community-based practice/rural) throughout central and western Pennsylvania within the health system’s network.

Demographics, clinical information, and administered treatments were compiled from tumor registry records. We categorized contextual factors to represent diverse backgrounds with different hospital setting (academic vs. community), clinical information (clinicopathology and hormone status), socioeconomic status based on the neighborhood deprivation index (NDI) as well as age and race to examine association between these factors and the DCIS treatments.

### Quality-of-life (QOL) survey

QoL survey was designed as a pilot project to look at the feasibility in collecting data from DCIS patients with diverse backgrounds and different socioeconomic statuses. The letter of invitation and consent forms for the QOL survey were sent to 130 patients who were randomly selected based on their treatments, sociodemographic characteristics, and treatment locations from the study cohort. All Black women alive (*n* = 69) in the study cohort were selected as the first batch. Next, for White and other races, every 5th patient in the list in each treatment group was selected. The half of these patients was selected from academic practice sites and the other half from community sites. If NDI and age were close to each other among those selected patients, patients were re-selected to balance age and NDI between patients. For those who consented, a quality-of-life questionnaire breast cancer module 23 (QLQ BR-23) assessing QoL specific to breast cancer [20] and qualitative assessment questions was administered via phone interview. The phone interview was planned for 45 min for each participant and performed between March 1 and April 30th in 2022.

The questionnaire, BR-23, includes 23 questions with the functional and symptom domains using five multi-item scales assessing body image, sexual functioning, systemic therapy side effects, breast symptoms, and arm symptoms. In addition, there are three single-item scales assessing for sexual enjoyment, future perspective, and being upset by hair loss [20].

The qualitative assessment questions were intended to examine treatment making process, satisfaction or regret with treatment decision, and factors affecting treatment decision-making [21].

### Data analyses

SAS 9.4 (SAS Institute, Cary, NC) and R 4.1.2. software were used for statistical analyses. Fisher’s two-sided exact test was used to compare DCIS treatments received between two groups: (1) Academic and community-based (non-academic) sites, (2) Black and White, (3) Neighborhood deprivation index (NDI) median level and extremes (> = 75 vs. < 75%), (4) Clinicopathologic grades (low/intermediate vs. high), (5) Hormone status (Estrogen Receptor positive vs. negative), and (6) Age groups (< = 50, 50–70, > 70 years old). Treatments were stratified into 4 different schemes: breast-conserving surgery, breast-conserving surgery with radiation therapy (RT), breast-conserving surgery with RT and endocrine/hormone therapy (HT), and mastectomy.

Median, minimum, and maximum scores for all 6 QoL domains with functional and symptoms scales in BR-23 were calculated. Scoring calculations for the functional and symptoms scales were based on the BR-23 scoring manual [20].

All scores were compared using Kruskal–Wallis test between treatment groups (breast-conserving surgery only, breast-conserving surgery with radiation, breast-conserving surgery with RT and HT, and mastectomy). Also, Mann–Whitney U test was used to compare all QOL scores between races and between hospitals. Kruskal–Wallis and Mann–Whitney U tests were used due to non-parametric distribution of data and compare non-normal distributions. BR-23 module has items examining systemic therapy/chemotherapy side effects [20]. However, as chemotherapy side effects including hair loss are not applicable for DCIS, we modified the items on systemic therapy to reflect hormone/endocrine therapy side effects (HT) and excluded the item “upset by hair loss” [20] (Appendix Table 10). Two-sided probability values of < 0.05 were considered statistically significant.

## Results

### Study population characteristics

A total of 941 patients who were diagnosed and treated for DCIS between 2018 and 2019 in UPMC-integrated network were identified. Among these, 29 patients were excluded from the study because they were on COMET trial (de-escalation with the omission of surgery). Of the remaining 912 patients, 506 patients were from academic sites and 406 patients from community sites. Median patient age was 63 years old (range: 24–90 years). Median NDI was 59% (range: 1–100%; 100% representing the worst NDI). 46 patients refused recommended RT and 80 patients refused recommended HT.

Table 1 shows the summary of the study patient characteristics in this study cohort.

**Table 1:** Patient characteristics

Number of patients	*n* = 912
Median age at diagnosis in 2018–2019 (range) Age distribution	63 (24–90)
	< = 50 years old; n = 166 (18.2%)
	50–70 years old; n = 547 (60%)
	> 70 years old; n = 199 (21.8%)
Race	Black: *N* = 71 (7.8%)
	White: *N* = 830 (91%)
	Other: *N* = 11 (1.2%)
Median NDI (Scale: 100% as the worst)	59% for All
	85.5% for Black only
Patients’ Treatment Location	*N* = 506 at Pittsburgh (Urban)
	*N* = 406 outside Pittsburgh (Rural)
Estrogen Receptor status	Positive: *n* = 707 (77.5%)
	Negative: *n* = 117 (12.8%)
	Unknown: *n* = 88 (9.7%)
Progesterone Receptor status	Positive: *n* = 635 (69.6%)
	Negative: *n* = 210 (23%)
	Unknown: *n* = 67 (7.4%)
Clinicopathologic Grade	Low: *n* = 86 (9.4%)
	Intermediate: *n* = 347 (38%)
	High: *n* = 342 (37.5%)
	Unknown: *n* = 137 (15%)
Treatments received	SHR: 37.5%
	SR: 12.9%
	Mastectomy: 25.2%
	S only: 12%
	SH: 12.4%
HT refusal	*n* = 80
RT refusal	*n* = 46
Death	*n* = 18 (*n* = 2; Black)

*S* lumpectomy, *H (HT)* hormone therapy, *R (RT)* radiation therapy

### Associations between factors and treatment received

1. Academic practice vs. Community-based sites:
  The proportion of patients who received RT was not significantly different between hospital settings (academic practice 48.6% vs. community sites 52.6%; *p* = 0.47). Also, most patients had consistent RT dose and fraction size regardless of hospital setting (hypo fractionated RT receiving 40.05 or 42.16 Gy in 15 or 16 fractions with or without boost). Appendix Table 7 summarizes the treatments administered between two hospital settings, academic/urban and community/rural sites.
  HT utilization rate differed between the two hospital settings: 62 and 74% of ER-positive patients received hormone therapy in academic and community sites, respectively (*p* = 0.0012). Thus, patients from community sites more often received hormone therapy than those in academic practices.
  Also, mastectomy rate on DCIS differed between the hospital settings. Academic practice performed mastectomy more often than community sites, for 29 and 20.4% of DCIS patients (*p* = 0.0045). Among all mastectomy patients, 70% had sentinel node biopsy and 5 patients (2%) had bilateral mastectomy. Also, 36% (84/231) of mastectomy patients received reconstruction. As a note, reconstruction rate among mastectomy patients was not significantly correlated to the different treatment locations (*p* = 0.09), age groups (*p* = 0.32), races (*p* = 0.76), and NDI median level (*p* = 0.31).
2. Age groups (< = 50, 50–70, > 70 years old):
  Compared to age groups for < = 50 and 50–70 years old, > 70-year-old group were less likely to receive RT (*p* < 0.001). For HT utilization, age groups were not significantly different (*p* = 0.07). In addition, <  = 50-year-old age group had higher rate of mastectomy than > 50 years old (37 vs. 23%; *p* < 0.001).
3. Race (Black vs. White):
  There was no difference in treatments received between the Black and White groups (*p* = 0.5, 0.69, and 0.09 for RT, HT, and mastectomy, respectively).
4. NDI (Socioeconomic status):
  There was no difference in treatments received at median NDI level (> 59% vs. < = 59%). In addition, we examined the treatment difference at the highest deprivation (> 75% vs. < = 75%) and no difference between these groups was found as well in terms of treatments received. Of note, there was a significant difference in NDI between the Black and White groups. (85.5% vs. 57%; *p* < 0.0001).
5. ER status (ER positive vs. Negative):
  ER-positive status was strongly correlated to receiving HT (*p* < 0.0001).
6. Clinicopathologic grades (Low/Intermediate vs. High):
  High-grade DCIS group was associated with higher RT utilization than low/intermediate-grade group (*p* < 0.0001).

Table 2 summarizes the above results.

**Table 2:** Associations between factors and treatments received

Characteristics	Association with treatments received
Age	RT: > 70 years old had less RT than other groups (53 vs 71%; *p* < 0.001), Mastectomy: < = 50 years old vs. > 50 years old (37 vs. 23%;*p* < 0.001)
Race	No
NDI	No
Treatment locations (academic/urban vs. community/rural)	(1) RT: no (48.6 vs. 52.6%;*p* = 0.47)
	(2) Mastectomy: Yes (29 vs. 20.4%;*p* = 0.0045)
	(3) HT: Yes (62 vs. 74%;*p* = 0.0012)
ER status (Positive vs. Negative)	ER-positive status receiving higher HT (*p* < 0.0001)
Clinicopathologic grade (Low/intermediate vs. high)	High grade utilizing higher RT than low/intermediate grade (*p* < 0.0001)

### Quality-of-life survey

42 out of 130 patients (32%) consented to participate in QOL survey interview. Among the 42 patients, QOL surveys via phone interview were completed for 38 patients. The Black patients were 37% (*n* = 14) of the QoL survey participants, and it was 20% of a total number of the Black patients in this study cohort (14 out of 71). Table 3 shows the characteristics of 38 participants who completed the phone interviews.

1. QOL scores between treatment groups (Surgery, surgery with RT, surgery with RT and HT, mastectomy):
  Scores between treatment groups were not significantly different except for HT items (Table 4).
2. QOL scores between races (White vs. Black):
  Black patients reported worse HT side effects than White patients (Table 5).
3. QOL scores between hospitals (Academic vs. community practices):
  There was no statistically significant difference between the hospital settings, but arm symptoms score trended toward worse outcomes for patients treated at community hospitals (*p* = 0.065; Table 6).
4. Qualitative assessment for decision-making and satisfaction in care (scale 1–5; 1-best; and 5-worst) (Appendix Table 8)
  (i) Resources for treatment decision: 31 participants said that the surgeon and the medical team were the most helpful resources to determine treatment options (82%) and family and friends were the second highest (46%).
  (ii) Knowledge learned for treatments and side effects: 29 participants responded that they understood all the treatment-related side effects very well (76%; scale 1 and 2). But 9 participants (24%) were not well informed of treatments and their side effects.
  (iii) Satisfaction with decision for treatments: 2 participants had decisional regret due to HT and RT treatment-related side effects.
  (iv) Satisfaction with the current health status/life: 33 participants (87%) were happy with the current health status and were satisfied with their lives (scale 1 and 2).
5. Qualitative assessment to examine factors affecting the treatment decision (Appendix Table 9)

**Table 3:** Quality-of-Life survey participants’ characteristics

Consented for the participation	*N* = 42 (42/130 = 32%)
Completion of the phone interview	*N* = 38
Median age (range)	56.5 (42–80)
Race	Black: *n* = 14, White: *n* = 24
Median NDI (range)	61% (24–100%)
Treatment location	Academic/urban: *n* = 24
	Community/rural: *n* = 14
Treatment received (n)	SHR (19), Mastectomy (9; reconstruction 5), SR (6), S (4)
RT	3–4 weeks
HT, RT refusal	6 (HT: n = 5, HT and RT: *n* = 1)

*RT* or *R* radiation therapy, *HT* or *H*: hormone/endocrine therapy, *S*: lumpectomy surgery

**Table 4:** QOL scores between treatment groups (Surgery with RT and HT, Surgery with RT, Mastectomy, and Surgery only)

BR-23 domains	*n*	SRH		SR		Mastectomy		S	*p*-value
		Median (min, max)	*n*	Median (min, max)	*n*	Median (min, max)	*n*	median(min, max)	
HT side effects	19	45.8 (0, 87.5)	6	16.7 (4.2,45.8)	9	8.3 (4.2, 54.2)	4	12.5 (0,12.5)	0.0017*
Arm symptoms	19	0 (0,100)	6	16.7 (11.1, 44.4)	9	0 (0,44.4)	4	0 (0,11.1)	0.2061
Breast symptoms	19	16.7(0,66.7)	6	25 (0,33.3)	9	8.3(0,33.3)	4	0 (0,33.3)	0.5153
Body Image	19	16.7(0,50)	6	8.3 (0,91.7)	9	16.7(0,58.3)	4	0.0	0.2283
Future perspective	19	33.3(16.7, 100)	6	33.3 (0,50)	9	16.7(0,83.3)	4	16.7(0,33.3)	0.5754
Sexual function/ enjoyment	19	66.7(11.1, 100)	6	61.1(22.2, 100)	9	44.4 (11.1,100)	4	100 (33.3,100)	0.4937

Higher QOL scores corresponded to worse impact on quality of life in these analyses

*P < = 0.05, statistically significant

*QOL* quality of life, *HT(H)* hormone therapy/endocrine therapy, *S* surgery (lumpectomy), *SRH* surgery, radiation therapy, and hormone/endocrine therapy, *SR* surgery and radiation therapy, *R (RT)* radiation therapy

Bold indicates SRH group has worse HT side effects than other treamtent groups

**Table 5:** QOL scores between races (White vs. Black)

BR-23 item	*n*	Whites		Blacks	*p*-value
		Median (min, max)	*n*	Median (min, max)	
HT side effects	24	12.5 (0,75)	14	47.9 (16.7, 87.5)	0.0041*
Arm symptoms	24	11.1 (0,44.4)	14	5.6 (0,100)	0.8928
Breast symptoms	24	16.7(0,50)	14	8.3 (0,66.7)	0.1803
Body Image	24	8.3 (0,91.7)	14	8.3 (0,41.7)	0.9275
Future perspective	24	33.3 (0,50)	14	16.7(16.7,100)	0.9843
Sexual function/enjoyment	24	66.7 (22,100)	14	66.7 (11.1,100)	0.8933

Higher QOL scores corresponded to worse impact on quality of life in these analyses

*P < = 0.05, statistically significant

*QOL* quality of life, *HT* hormone therapy/endocrine therapy

Bold indicates Black women had worse HT side effects than White women

**Table 6:** QOL scores between hospitals (Academic vs. community practices)

BR-23 item	Academic practice sites		Community sites		*p*-value
	*n*	median (min, max)	*n*	median (min, max)	
HT side effects	24	20.8 (0,87.5)	14	39.6 (48,62.5)	0.3011
Arm symptoms	24	0 (0,44.4)	14	11.1 (0,100)	0.0645
Breast symptoms	24	8.3 (0,33.3)	14	25 (0,66.7)	0.1066
Body Image	24	0 (0,50)	14	16.7 (0,91.7)	0.0956
Future perspective	24	33.3 (0,100)	14	25 (0,83.3)	0.5014
Sexual function/enjoyment	24	66.7 (11.1,100)	14	66.7 (44.4,100)	0.0983

Higher QOL scores corresponded to worse impact on quality of life in these analyses

*P* < = 0.05, statistically significant

*QOL* quality of life

*HT* hormone therapy/endocrine therapy

Participants ranked items provided for considering the most important/influential factor when deciding treatments. 82% of participants ranked the physician’s recommendation as the most influential factor to consider treatments. Fear of recurrence (74%) and treatment-related side effects (38%) were the next highly ranked items. Notably, there were no different pattern or items found between races or age groups or treatment groups in the decision-making process and rankings for influential factors in decision (Appendix Table 9).

## Discussion

Our study demonstrated that DCIS treatment patterns were not different between races, socioeconomic status, and different regions in the UPMC-integrated health system. Strikingly, RT-administered dose and utilization rate were consistent regardless of age groups, race, NDI, and treatment locations. More importantly, this study demonstrated the feasibility in collecting DCIS-specific QoL and treatment decision-making process for patients with diverse backgrounds and socioeconomic status that have been limitedly available. The survey participation from the Black women was 20% of a total number of the Black women in this study cohort and 37% among all survey participants.

Nationwide clinical databases, including SEER and NCDB, as well as multiple institutions, have shown substantial difference in practice patterns for DCIS based on geographic and/or racial differences rather than on patient-level variation. [15, 22–24] Also, the report from Kaiser Permanente community-based health plan data for DCIS showed variations in DCIS treatment patterns by races and regions in the same integrated health system [25].

The major contributing factor to the current results showing less variation in treatment patterns could be due to clinical pathways and peer review policies implemented in all the UPMC-integrated health plan sites to guide DCIS treatment. Contrary to recent reports, including from SEER and NCDB, for DCIS treatments, RT utilization rate in our cancer center network was 50% (Table 1), lower than the commonly reported RT utilization rate (70%), [26–28] likely reflecting that standardized care with clinical pathways may reduce overtreatment with RT. However, high-grade DCIS patients received more RT than low/intermediate patients, in line with national trends and randomized trials that show high rates of recurrence without RT in high-grade DCIS [29, 30].

Our network mastectomy rate for DCIS in 2018–19 was comparable to recent SEER data that was compiled for cohort in 2000–2013 (25%) [28], showing that the mastectomy utilization remains largely unchanged over time. Interestingly, another recent study using NCDB data showed that bilateral mastectomy for patients with DCIS under the age of 50 years has increased significantly from 2004 to 2016 (11–27%), while unilateral mastectomy rate has been consistent over time. Moreover, in younger age women (< = 40 years old), bilateral mastectomy rates (40%) surpassed lumpectomy rates (35%) [31]. However, our cohort did not show such a high rate of bilateral mastectomy. The unilateral mastectomy rate for women under 50-year-old age had higher rates of mastectomy than > 50-year-old age group, which is similar to NCDB findings. Another interesting finding from the current study was variation in mastectomy rates between academic and community-based practices, with academic hospitals having higher mastectomy rates (29%) than community sites (20.4%) (*p* = 0.0045). A likely explanation includes disease characteristics as patients with diffuse, multicentric disease are more often managed at the academic practice locations with more integration of additional surgical subspecialties such as plastic surgery is more readily available.

Also, there was a variation of utilization in HT between the hospitals and about 9% of this study cohort (*n* = 80) refused HT at the time of treatment decision. In fact, there is no clear consensus for the use of HT. Adverse effects and poor adherence are common. Moreover, early termination of endocrine therapy for patients with DCIS has not affected local control or overall survival [26, 32, 33]. These findings underscore the need for better patient–physician decision-making processes that incorporate consideration of HT benefits and harms and in line with the most recent study by Levy J et al. [34].

For the QOL survey, only HT side effects negatively affected QOL scores between treatment groups (Table 4). HT/endocrine therapy has known worse QOL with poor adherence, which our study also demonstrated. Interestingly, Black patients reported worse QOL with HT compared to White patients. Schleinitz et al. and Hu et al., [16, 35] reported the similar results as ours showing that Black women noted lower quality of life with HT during breast cancer treatments.

For our qualitative assessment interview, most participants were satisfied with the treatments received and the care they received. The most trusted resource for their treatment decision-making was physician (and the medical team). Decisional regret around DCIS treatment decisions was quite low with 2 participants among 38 participants. In addition, the most influential factors for considering treatments were also the physicians’ recommendation followed by fear of recurrence and treatment side effects. Our QoL survey and qualitative assessment interview results showed that there was relatively less variation in QoL scores in different treatment groups, while a couple of studies reported that patients who underwent mastectomy with sentinel biopsy had worse QoL scores as compared to patients who underwent lumpectomy. [36, 37] The fear of disease recurrence was reported as the most important factor in affecting the QoL and perhaps over treatments in DCIS. [7, 38–40] The present study similarly showed that the likelihood of recurrence was one of high ranked items for factors affecting the decision-making, but interestingly the highest ranked item for affecting treatment decision-making in our study participants was the physician and the medical team’s recommendation, emphasizing the importance in the care providers’ role in communication, well informed, and shared decision-making process with patients [41]. Interestingly, there was not a noticeable difference in QoL for body image domain between patients with and without reconstruction and body image was not a highly ranked item in treatment decision-making either. As stated earlier, clinical pathway and peer review could play a critical role for physician and medical team to inform and help the patients for decision-making better. In an era of value-based cancer treatment, reductions in overtreatment and less variation in practice pattern may lead to cost saving or more cost-effective treatment from an economic perspective [42]. The COMET trial is currently conducted for comparing active surveillance to standard therapy for patients with low- risk DCIS [43]. This trial will provide guidance in managing low-risk DCIS by observation without standard therapies.

One of the limitations of this study is that 60% of patients of the academic practice centers were from one single center, a Women’s specialty hospital. Another limitation was retrospective study design for QOL surveys and qualitative assessments with small sample size (*n* = 38) that may not accurately capture all the survey items at the time of treatments and over time. For example, it is known that the RT could deteriorate QoL on patients with RT during treatment or up to 1-year post-RT, but the QoL becomes better and remains the same after 2 years. This study cohort’s treatments were finished in 2018–19, so the QoL surveys at the time of this study may not reflect the QoL during the RT. Also, the QoL measurement tool, while validated for invasive breast cancer, may not adequately measure the concerns of women with DCIS. The QLQ-BR-23 used to measure QoL in this study is appropriate for capturing breast cancer-related symptoms, although the nuance of DCIS concerns may not be fully captured. In addition, our cancer center network tumor registry does not include the information for comorbidities. Patients with significant comorbidities less likely to get mastectomy and would be better with adjuvant therapies, which limits treatment choices. Lastly, we did not examine insurance status or type for our study cohort. Our integrated health system has its own health plan which by internal network review, about 25–30% of the patients were covered by our own health plan.

In summary, DCIS treatment based on clinical pathways and rigorous peer review in our integrated health system network did not show chronically noted disparities in cancer care arising from social determinates of health. Also, our RT utilization rate was lower than national rates. HT use was variable across the network, and mastectomy rates was similar to national levels, unchanged over last two decades. Importantly, this study demonstrated feasibility in collecting QoL and qualitative assessment data for DCIS women with diverse backgrounds and various socioeconomic status, which has been scarce, and can be a framework for future prospective study.

Except for HT use, QoL scores in items using BR-23 module for different treatments between races and between treatment locations were not significantly different. Treatment decision-making process and factors affecting decision-making was mainly from the physician and the medical team’s recommendations regardless of age, race, treatment locations, and treatment groups, highlighting the importance in shared and informed decision-making between the patients and the care provider to reduce health disparity.

In future research, we will collect QoL from a larger sample of DCIS patients prospectively in different time points with planned oversampling of underserved, low-income women to elicit DCIS-specific QoL and investigate an impact of variation in practice patterns on the cost-effectiveness of DCIS treatment. 

## Data Availability

Enquiries about data availability should be directed to the authors.

## Appendix

See Tables 7, 8, 9, and 10.

**Table 7:** Treatments received in different hospital settings

Treatments	Academic/Urban	Community/Rural	Total (%)
SHR	167 (33%)	175 (43%)	37.50
SR	79 (15.6%)	39 (9.6%)	12.90
S (lumpectomy)	59 (11.7%)	50 (12.3%)	12
Mastectomy	147 (29%)	83 (20.4%)	25.20
SH	54 (10.7%)	59 (14.5%)	12.40
Total	506	406	912/100

*S* lumpectomy, *H* hormone therapy, *R* radiation therapy

**Table 8:** Qualitative assessment questionnaire for decision-making and satisfaction

Survey Items
How did you learn about the treatment options besides from your physician(s)? **1. Internet/TV, 2. Your own Literature search, 3. Friends/Family members who knew about DCIS, 4. Other (please list)**
Were you informed well from your physicians regarding the potential side effects of each treatment you received? **(very well-1, quite well-2, not very well-3, a little-4, Not at all-5)**
With any treatment, things may come up that you don’t expect. Looking back, how well do you think you understood the treatment you received before you started treatment? **(very well-1, quite well-2, not very well-3, a little-4, Not at all-5)**
Who/what was the most helpful resource about your treatment decision-making process? **1. Physician(s), 2. Internet/TV, 3. Your own Literature search, 4. Friends/Family members who knew about DCIS, 5. Other (Please list)**
Do you regret for your treatment choice **(Yes or No)** and why?
What facts of the DCIS treatments you wish you knew better before you made decision?
Are you content with/enjoying your current life? **(very much-1, quite -2, not so much-3, a little-4, Not at all-5)**

**Table 9:** Factors affecting the treatment decision

Ranking items for decision-making	Ranking final
Body image	9
The likelihood of disease recurrence	2
The complications and side effects of the treatments affecting physical, mental, and social well-being	3
What my physicians/medical team recommends for treatment?	1
What other women like me choose for treatment?	8
The number/length of treatment sessions	6
Length of recovery period	4
Time off from work or any activities	6
Insurance coverage	11
Out of pocket expenses and financial difficulties	10
Travel/logistics	12
Burden on or receiving support from spouse/family/care givers	5
Other (Please list)*	

*5 participants mentioned all various issues but did not rank

**Table 10:** HT side effects with modified items on systemic therapy domain in BR-23

31	Did you gain /lose weight?
32	Did you have vaginal dryness or/ and discharge?
33	Did you have night sweats/difficulty in sleeping?
34	Have you had mood swings /irritable/feeling bloated?
35	Did you have joint pain?
36	Did you feel ill or unwell in general?
37	Did you have hot flushes?
38	Did you feel lack of energy or difficulty in concentrating?
